# Effect of antagomir-22-3p treatment on skeletal muscle growth in intrauterine growth-restricted lambs

**DOI:** 10.3389/fmolb.2025.1547182

**Published:** 2025-05-16

**Authors:** S. K. Duckett, M. A. Greene, A. N. S. Udoka, R. R. Powell, T. F. Bruce, J. L. Klotz

**Affiliations:** ^1^ Department of Animal and Veterinary Sciences, Clemson University, Clemson, SC, United States; ^2^ Clemson Light Imaging Facility, Clemson University, Clemson, SC, United States; ^3^ Department of Bioengineering, Clemson University, Clemson, SC, United States; ^4^ Forage-Animal Production Research Unit, United States Department of Agriculture-Agricultural Research Service (USDA-ARS), Lexington, KY, United States

**Keywords:** sheep, intrauterine growth-restricted, muscle, microRNA, antagomir-22-3p

## Abstract

**Introduction:**

MicroRNAs (miRNAs) play a crucial role in regulating gene expression and muscle development. Previous research identified miR-22-3p as being differentially regulated during muscle hypertrophy, and *in vitro* experiments found that antagomir-22-3p enhanced satellite cell proliferation. The objective of this study was to evaluate the *in vivo* vascular injection of antagomir-22-3p into the lateral saphenous vein and its effect on miRNA and histone deacetylase (HDAC) family mRNA expression in intrauterine growth restriction (IUGR) lambs.

**Methods:**

Pregnant ewes (n = 18) carrying twins were fed either 100% of NRC (CON) or were nutrient-restricted to 60% NRC (NR) from gestational d 86 until parturition. On d 12 of age, NR lambs (n = 8) were randomly selected and given a systemic injection of antagomir-22-3p (440 µg/lamb) into the lateral saphenous vein of the right leg (NR-ANT22) for three consecutive days. CON lambs (n = 8) were also randomly selected and received a sham injection of phosphate-buffered saline (PBS) into the lateral saphenous vein of the right leg (CON-SHAM). Blood samples were collected from each lamb weekly to monitor circulating miR-22-3p expression. Muscle samples were collected 24 days post-injection to assess miR-22-3p levels and mRNA expression of potential miRNA targets.

**Results and discussion:**

Cell-free circulating miR-22-3p expression was more downregulated (P < 0.05) in NR-ANT22 lambs compared to that in CON-SHAM on d 14 after injection. On d 21 after injection, miR-22-3p expression in plasma tended to be more downregulated (P = 0.08) in NR-ANT22 compared to CON-SHAM. Lamb body weight and muscle weights at harvest were similar between the NR-ANT and CON- SHAM treatment groups. In the semitendinosus (ST) muscle, miR-22-3p expression was more downregulated (P < 0.05) in NR-ANT22_R ST (treated) lambs compared to that in NR-ANT22_L ST (non-treated) and CON-SHAM ST. In the heart and semimembranosus (SM) muscles, the expression of miR-22-3p was more downregulated (P < 0.05) in NR-ANT22 compared to that in CON-SHAM. In the gastrocnemius muscle, miR-22-3p expression remained unchanged (P > 0.05). The number of types I, IIa, and IIax muscle fibers were greater (P < 0.05) in NR-ANT22 lambs than those in CON-SHAM, whereas the number of type IIx fibers was greater (P < 0.05) in CON-SHAM lambs. NR-ANT22 treatment appears to promote a shift toward more oxidative muscle fiber metabolism. The systemic injection of antagomir-22-3p downregulated miR-22-3p expression in circulation and muscle tissues, which, in turn, altered the expression of HDAC/SIRT genes involved in muscle fiber type conversion and hypertrophy.

## 1 Introduction

Intrauterine growth restriction (IUGR) alters muscle development and hinders postnatal muscle growth in lambs (Symonds et al., 2010; Reed et al., 2014; Hoffman et al., 2016; Hoffman et al., 2017; Yates, et al., 2016; Gauvin et al., 2020; Greene et al., 2019; Greene et al., 2022b). Maternal nutrient restriction or overfeeding of ewes during mid to late gestation can alter the ratio of secondary to primary muscle fibers and reduce the number of PAX7-positive cells (Gauvin et al., 2020). Intrauterine growth-restricted lambs have myoblasts with reduced proliferation capacity *in vitro* (Yates et al., 2014). Pillai et al. (2016) reported that multipotent mesenchymal stem cells, isolated from bone marrow, had lower proliferation rates and altered metabolism in lambs from ewes fed either restricted or excessive nutrient levels during gestation. Further research is needed to examine potential technologies that could enhance postnatal muscle growth in IUGR lambs by stimulating myoblast proliferation and promoting muscle mass development.

MicroRNAs (miRNAs) are small, non-coding RNAs that can regulate muscle stem cells or satellite cells to alter muscle growth (Horak et al., 2016; Koopmans et al., 2023). Transcriptomic analyses of small RNAs identified six miRNAs (miR-652, -22-3p, -127, -133a, -129-1-3p, and -615) that were differentially expressed in IUGR fetuses exposed to ergot alkaloids in endophyte-infected tall fescue (Greene et al., 2019). Developmental assessment of changes in miRNA transcriptome during muscle development showed that differential expression of miRNAs was most pronounced during the transition from late gestation to early postnatal growth in lambs, with miR-22-3p showing the greatest upregulation during this time period (Greene et al., 2022a).

The downregulation of miR-22-3p using antagomirs increased satellite cell proliferation *in vitro* (Greene et al., 2023). Direct injection of antagomir-22-3p in the longissimus muscle of IUGR lambs altered miR-22-3p expression and histone deacetylase 4 (HDAC4) expression and converted muscle fiber type to a more oxidative state (Greene et al., 2023). miRNAs are being evaluated as potential therapeutic treatments for cancer and muscle wasting in humans and have the potential to accelerate muscle growth in livestock animals. The objective of this study was to examine the effects of systemic injection of antagomir-22-3p on circulating and tissue expression of miR-22-3p and changes in muscle fiber type in IUGR lambs during early postnatal growth.

## 2 Materials and methods

The use of animals for this experiment was approved by the Clemson University Institutional Animal Care and Use Committee (AUP2022-0472).

### 2.1 Animals

Mature Suffolk ewes (n = 24) were randomly divided into two groups (n = 12/group) and synchronized using a controlled intravaginal drug release device (EAZI-BREED™ CIDR^®^; Zoetis). Ewes were mated to a Texel ram (Texel-Muscled, GeneSeek). Pregnancy detection was performed using real-time ultrasound (BCF Easi-Scan Portable Ultrasound, BCF Technologies, Rochester, MN, United States), and ewes estimated to be carrying twins (n = 18) were used for the study. Ewes were randomly allotted to one of two nutritional feeding treatments: nutrient-restricted (n = 9; 60% NRC) from d 86 to parturition in order to induce IUGR (NR; Hoffman et al., 2016) or non-restricted controls (n = 9; CON; 100% NRC). Ewes carried to term, and lamb birth weight was recorded. One ewe on the CON diet delivered lambs prematurely, and they were not used for this study. One ewe on the NR diet delivered twin lambs, but one died shortly after birth due to dystocia. This resulted in 16 ewes (n = 8 per treatment) with twin lambs for this study. Litter size in sheep impacts birth weight and postnatal growth rates (Gardner et al., 2007). After birth, all ewes were fed a CON diet, and no further nutrient restriction occurred during the lactation period.

Lambs were maintained with their dams, and all ewes were fed at normal intake levels during lactation. At 14 days postnatal, one lamb from each NR ewe with twins was randomly assigned to receive a d 0 postnatal miRNA treatment consisting of antagomir-22-3p (NR-ANT22; 440 µg/lamb; Creative Biogene) mixed with phosphate-buffered saline (PBS) and injected into the right lateral saphenous vein. One lamb from each CON ewe with twins was randomly assigned to a postnatal PBS sham (CON-SHAM) injection into the right lateral saphenous vein. Injections of ANT22 or SHAM were delivered using an intravascular delivery method to the hindlimb, as described by Hagstrom et al. (2004), but with slight modifications on the location of injection due to the use of young lambs in this study. ANT22 (n = 8) or SHAM (n = 8) was injected into the lateral saphenous vein of the right hind leg for three consecutive days (Krützfeldt et al., 2005; Hagstrom et al., 2004).

Krützfeldt et al. (2005) reported that injecting antagomir-122 for three consecutive days downregulated miR-122 expression for 23 days after the last injection in mice. For injections, a blood flow restriction band was placed on the injected leg near the hip to reduce the blood flow to 60% of normal and was maintained for 5 min to increase the uptake of ANT22 or SHAM into the plantar muscle group (Hagstrom et al., 2004). After 24 days from the last injection, all lambs were harvested, and individual muscle samples were collected immediately and frozen in liquid nitrogen for subsequent RNA extraction and muscle fiber histology. For the semitendinosus (ST) muscle, samples of muscle tissues were collected from the right (treated) leg (NR-ANT22_R), left (non-treated) leg (NR-ANT22_L), and right leg (CON-SHAM) for comparisons of miR-22-3p expression.

### 2.2 Cell-free circulating RNA

Blood samples were collected weekly from the lateral saphenous vein of the injected leg into EDTA tubes, both prior to and following injections, to monitor circulating concentrations of miR-22-3p. Tubes were centrifuged at 2000 × g for 20 min at 4°C, and plasma was removed. Plasma was stored at −80°C until extraction was performed. RNA was extracted using a plasma/serum cell-free circulating RNA advanced purification kit (Norgen, 68200), according to the manufacturer’s protocol. RNA was then converted to cDNA using the TaqMan MicroRNA Reverse Transcription Kit (Thermo Fisher). TaqMan MicroRNA Assay Kits were used for the target miR-22-3p (assay no. 242214_mat; catalog no. 444886) and the housekeeping gene U6 (assay no. 001973; catalog no. 4427975). The TaqMan PCR Assay Kit and a QuantStudio 3 Real-Time PCR System were used to conduct qPCR, according to the manufacturer’s protocol.

### 2.3 Muscle mRNA expression

Muscle samples were crushed using liquid nitrogen. Total RNA was extracted from 100 mg of muscle tissue using the TRIzol reagent (Invitrogen, Thermo Fisher Scientific, Waltham, MA), according to the manufacturer’s recommendations. RNA was purified using the Norgen RNA Clean-up and Purification Kit (catalog no. 43200) and Norgen DNase Kit (catalog no. 25710). Total RNA was quantified using a NanoDrop spectrophotometer. Samples were stored at −80°C until the reverse transcription reaction.

For mRNA expression analysis, total RNA (1 µg) was converted to cDNA using QuantaBio qScript cDNA SuperMix (VWR) and stored at −20°C. qPCR was performed using SYBR green (PerfeCTa SYBR Green SuperMix, low ROX; QuantaBio), according to the manufacturer’s protocol, with a QuantStudio 3 Real-Time PCR System (Thermo Fisher). PrimerQuest™ tool (IDT; Coralville, IA) was used to design primers. The genes that were investigated were sirtuin 1 (*SIRT1*), sirtuin 3 (*SIRT3*), sirtuin 6 (*SIRT6*), histone deacetylase 1 (*HDAC1*), histone deacetylase 2 (*HDAC2*), histone deacetylase 3 (*HDAC3*), histone deacetylase 4 (*HDAC4*), histone deacetylase 5 (*HDAC5*), histone deacetylase 6 (*HDAC6*), histone deacetylase 7 (*HDAC7*), histone deacetylase 9 (*HDAC9*), and histone deacetylase 11 (*HDAC11*). Eukaryotic transcription initiation factor 3 subunit k (EIF3K) and ubiquitously expressed prefoldin-like chaperone (UXT) were used as housekeeping genes (Greene et al., 2023). The geometric means of EIF3K and UXT were calculated and used for gene normalization. The 2^−∆∆CT^ method was used to normalize and calculate fold change from control (Livak and Schmittgen, 2001), and results are expressed as log2 fold change (log2FC).

### 2.4 Muscle fiber histology

Semimembranosus (SM) muscle from the left leg (treated) of each lamb was collected at harvest, cryopreserved in optimal cutting temperature, and cryosectioned, as described in Greene et al. (2022). Two tissue sections per animal were used for types I, IIa, and IIx myofiber typing. Cryosections of muscle samples were stained to identify types I, IIa, and IIx myofibers using the following primary antibodies: MHC-slow type 1 mouse IgG2b (Developmental Studies Hybridoma Bank [DSHB], Cat# BA-F8, RRID:AB_10572253), MHC-type IIa mouse IgG1 (DSHB Cat# SC-71, RRID:AB_2147165), and MHC-type IIx mouse IgM (DSHB, Cat# 6H1, RRID:AB_1157897). The following secondary antibodies were also used: Alexa Fluor 647 goat anti-mouse IgG2b (Thermo Fisher Scientific, Cat# A-21242, RRID:AB_2535811), Alexa Fluor 546 goat anti-mouse IgG1 (Thermo Fisher Scientific, Cat# A-21123, RRID:AB_2535765), and Alexa Fluor 488 goat anti-mouse IgM, (Thermo Fisher Scientific, Cat# A-21042, RRID: AB_2535711). Stained muscle sections were mounted in ProLong Gold (Cat #P36939, Invitrogen), and samples were imaged using a Leica DMi8 widefield microscope system (Leica Microsystems, Buffalo Grove, IL). To image proteins stained with Alexa Fluor 488 (Type IIx fibers, depicted in green), we used a GFP filter cube (Ex/Em 455–495/505–555 nm); to image proteins stained with Alexa Fluor 546 (Type IIa fibers, depicted in red), we used a Cherry filter cube (Ex/Em 540–580/592–668 nm); and to image proteins stained with Alexa Fluor 647 (Type 1 fibers, depicted in magenta), we used a Y5 filter cube (Ex/Em 600–660/662–738 nm). Four images per animal were used to assess the cross-sectional area and number of types I, IIa, and IIx muscle fibers using ImageJ software (NIH, https://imagej.nih.gov/ij/docs/guide/146-1.html) by a team of trained individuals.

### 2.5 Statistical analyses

Ewe body weight, body condition score, lamb body weight, and circulating miRNA expression were analyzed using the mixed procedure of SAS (SAS 9.4, Cary, NC) with treatment, time, and two-way interaction as fixed effects in the model. For lamb body weight, lamb sex was evaluated as a covariate in the model but was non-significant (P > 0.05) and removed from the final model. Tissue data were analyzed using ANOVA with treatment included as a fixed effect in the model (SAS 9.4). For histology, muscle fiber types and cross-sectional area were analyzed using the mixed procedure in SAS to compare NR-ANT and CON-SHAM treatments, with section and image included as random effects in the model. The ewe/lamb was the experimental unit. Least squares means were generated and separated with a protected least significant difference test. Statistical significance was determined at P < 0.05, and trends were considered at P < 0.10.

## 3 Results

**FIGURE 1:**
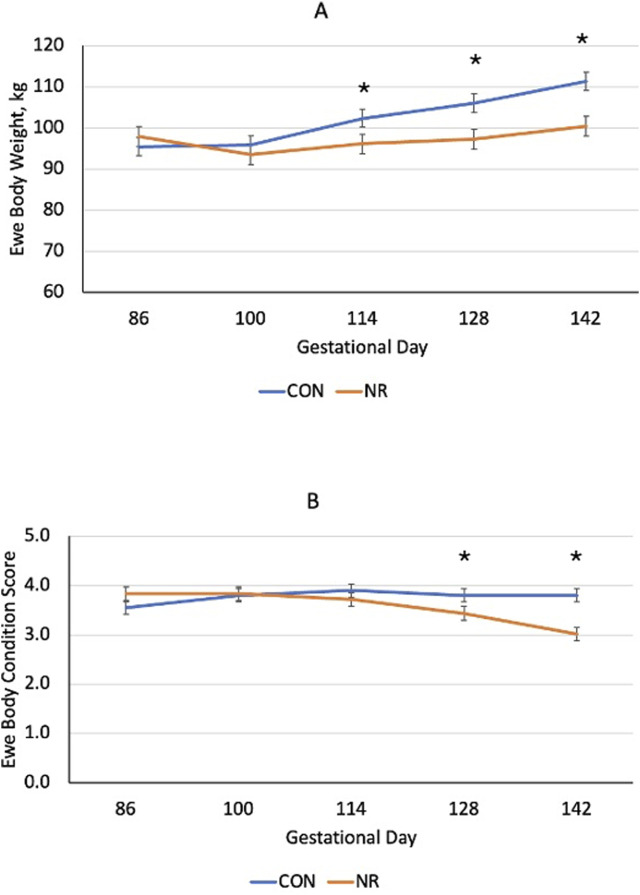
Effects of nutrient restriction (NR; 60% NRC) versus control (CON; 100% NRC) on ewe body weight **(A)** and body condition score [**(B)**; 1 = thin to 5 = obese] during late gestation (gd 85 to parturition). Standard error bars represent the standard error of the mean *(P < 0.05).

**FIGURE 2:**
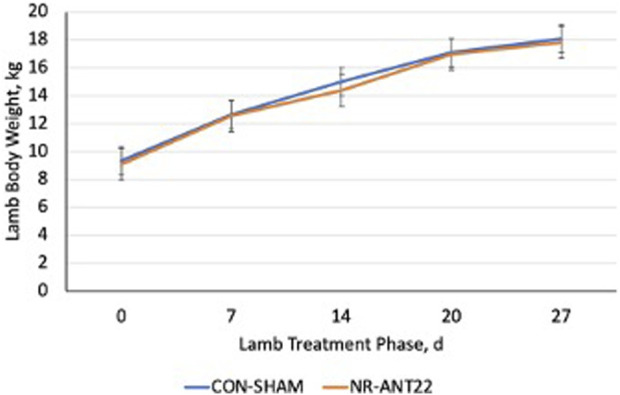
Effects of antagomir-22-3p treatment (NR-ANT22) compared to those of control (CON- SHAM) on lamb body weight during the miRNA treatment phase. Lamb body weight did not differ by treatment (P > 0.05). Standard error bars represent the standard error of the mean.

Nutrient restriction (60% of NRC) during late gestation more heavily altered (P < 0.041) body weight gain in ewes over time compared to that in controls. Body weight of NR ewes did not change from gestational day (gd) 86 to 142, whereas CON ewes gained 15.8 kg of body weight during this time (Figure 1A). Body condition scores of the ewes increased (P < 0.0051) over time in CON ewes (Figure 1B), but NR ewes lost body condition scores over time. Lamb birth weight did not differ (P = 0.82) between NR and CON ewes and averaged 4.32 kg. Lamb sex was included as a covariate but was non-significant (P = 0.80) and removed from the statistical model. Lamb body weight during the postnatal treatment phase did not differ (P = 0.99) over time for NR-ANT22 and CON-SHAM treatments (Figure 2).

**FIGURE 3:**
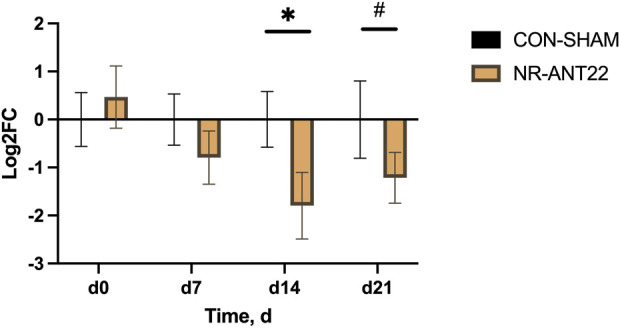
Effects of antagomir-22-3p treatment (NR-ANT22) compared to those of control (CON- SHAM) on circulating, cell-free miR-22-3p in lambs during the miRNA treatment phase. Standard error bars represent the standard error of the mean Significance is denoted as follows: *(P < 0.05) and #(P < 0.10).

Circulating cell-free miRNA expression of NR-ANT22 and CON-SHAM lambs is shown in Figure 3. There was an interaction between time and treatment (P < 0.05). Circulating miR-22-3p expression was downregulated in NR-ANT22 on d 14 (P < 0.05) and tended (P < 0.10) to be downregulated on d 21 on treatment compared to that in CON-SHAM. Our results indicate that circulating miRNA relative expression levels can be monitored to assess the efficacy of treatment. The greater reduction in expression on d 14 may indicate that this was the time when the greatest difference occurred.

Individual muscles were dissected from both the right leg (treated side) and left leg (non-treated side). Muscle weights did not differ (P > 0.05) between sides within the same lamb nor did they differ in the treated leg between NR-ANT22 and CON-SHAM groups.

**FIGURE 4:**
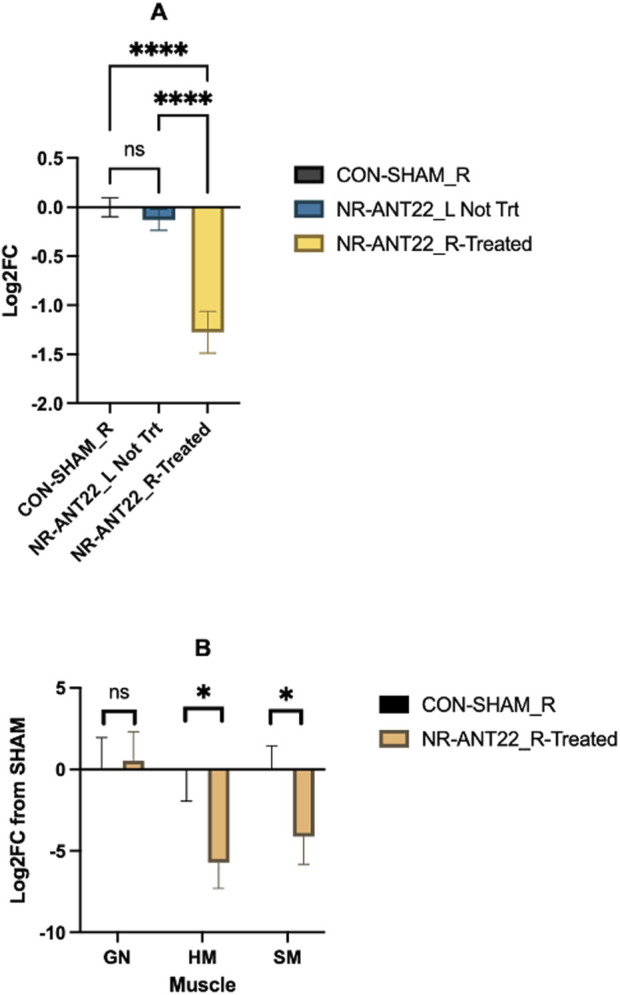
Effects of antagomir-22-3p-treated semitendinosus (ST) muscle (NR-ANT22_R treated) compared to those of NR-ANT22_L non-treated ST or control (CON- SHAM_R) on miR-22-3p expression in lambs at the end of the miRNA treatment phase **(A)**. Effects of antagomir-22-3p-treated ST (NR-ANT22_R Treated) compared to control (CON-SHAM_R) ST on miR-22-3p expression in lambs at the end of the miRNA treatment phase **(B)**. Muscle abbreviations: GN, gastrocnemius; HM, heart muscle; SM, semimembranosus; ST, semitendinosus. Standard error bars represent the standard error of the mean; significance is denoted as ****(P < 0.001) and *(P < 0.05).

In the ST muscle, miR-22-3p expression was more downregulated (P < 0.05) in NR-ANT right ST (treated) compared to that in NR-ANT22_L ST (non-treated) and CON-SHAM_R ST (Figure 4A). miR-22-3p expression did not differ between CON-SHAM_R and NR-ANT22_L ST (non-treated), indicating that antagomir-22-3p treatment downregulated miR-22-3p regardless of ewe treatment (NR or SHAM). The expression of miR-22-3p in muscle tissues on d 24 post-injection showed the greater downregulation (P < 0.05) of miR-22-3p in heart and semimembranosus muscles but not in gastrocnemius muscles of NR-ANT22 compared to that of CON-SHAM (Figure 4B). Hagstrom et al. (2004) found that intravenous delivery of naked plasmid DNA via the saphenous vein into the leg muscles was greater than intramuscular delivery. They also found that muscles closest to the injection site had the greatest number of positive staining cells for transgene expression.

**FIGURE 5:**
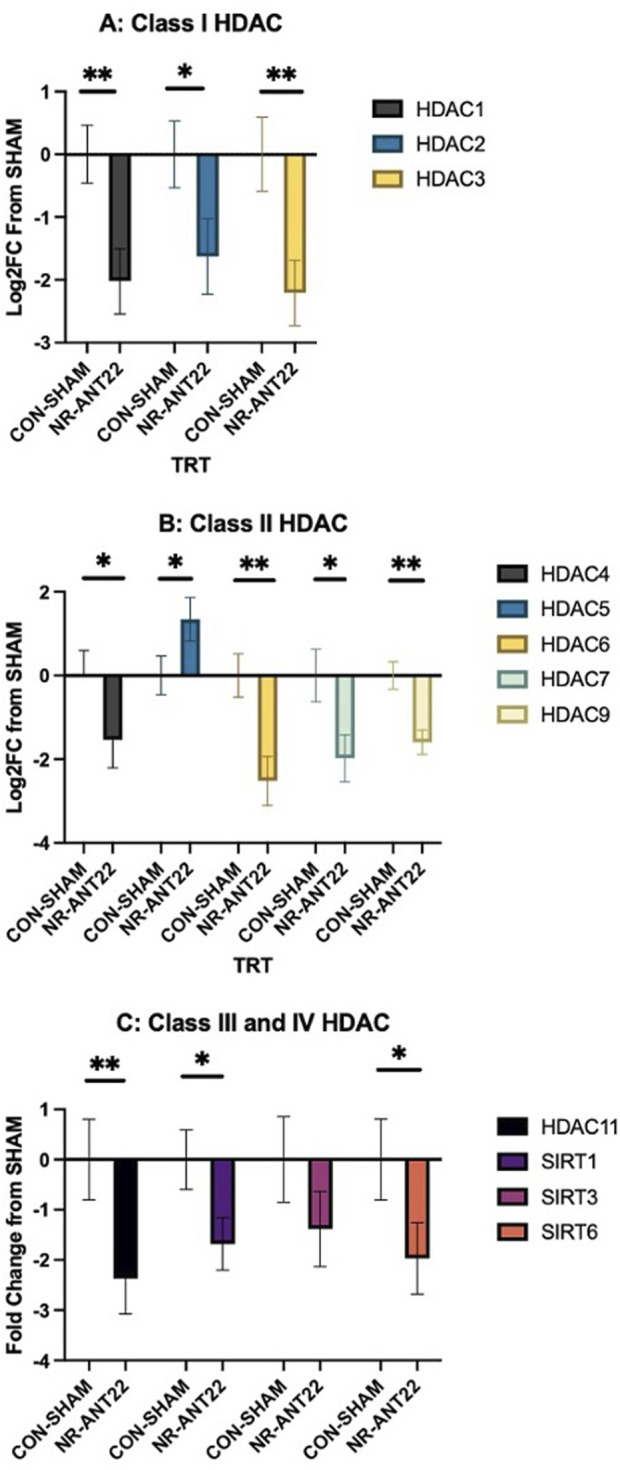
Effects of antagomir-22-3p treatment (NR-ANT22_R) compared to those of control (CON- SHAM_R) on the mRNA expressions of semimembranosus muscle HDAC classes I **(A)**, II **(B)**, III, and IV **(C)** in lambs during the miRNA treatment phase. Standard error bars represent the standard error of the mean; significance is denoted as ** (P < 0.01) and *(P < 0.05).

Targets of miR-22-3p are predicted to include HDAC4 and SIRT1 (Greene et al., 2023). mRNA expressions of the HDAC and SIRT family members are shown in Figure 5 for semimembranosus muscle. Class I HDAC members (HDAC1-3) were all more downregulated (P < 0.05) in NR-ANT22 semimembranosus muscle compared to those in CON-SHAM (Figure 5A). Class II HDAC members (HDAC4, HDAC6, HDAC7, and HDAC9) were more downregulated in NR-ANT22 semimembranosus muscle compared to those in CON-SHAM (Figure 5B). HDAC5 expression was more upregulated (P < 0.05) in NR-ANT22 semimembranosus muscle compared to that in CON-SHAM (Figure 5C). Class III (SIRT1 and SIRT6) and class IV HDAC (HDAC11) members were more downregulated (P < 0.05) in NR-ANT22 compared to those in CON-SHAM in the semimembranosus muscle. SIRT3 expression did not differ between NR-ANT22 and CON-SHAM. The HDAC family is involved in skeletal muscle metabolism and oxidative fiber phenotype activation (Molinari et al., 2023).

**FIGURE 6:**
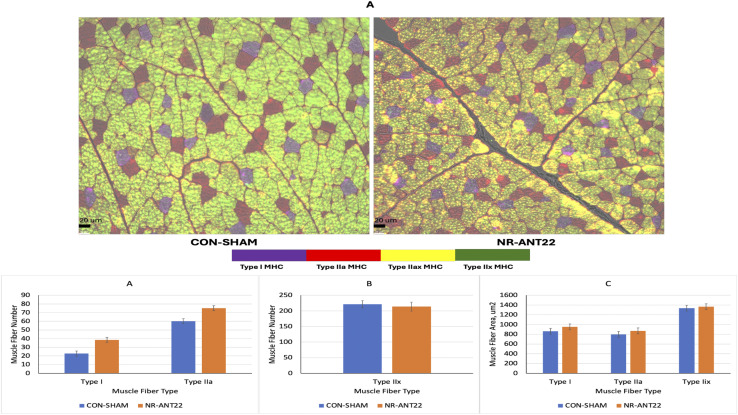
Effects of antagomir-22-3p treatment (NR-ANT22_R) compared to those of control (CON- SHAM_R) on types I, IIa, IIax, and IIx myofiber images **(A)** of muscle fibers from lambs at the end of miRNA treatment, muscle fiber number **(B)**, and muscle fiber cross-sectional area **(C)**. Standard error bars represent the standard error of the mean. Significance is denoted as ***(P < 0.001) and *(P < 0.05).

Muscle fiber type was determined with immunohistochemistry and antibodies for types I, IIa, and IIx heavy myosin chains (Figure 6A). The number of types I, IIa, and IIax muscle fibers was greater (P < 0.05) in NR-ANT22 than those in CON-SHAM (Figure 6B). The number of Type IIx muscle fibers was greater (P > 0.05) in CON-SHAM than that in NR-ANT22. The areas of the muscle fibers of types I, IIa, or IIx did not differ (P > 0.05; Figure 6C). The shift toward more oxidative fiber types is consistent with our previous experiments examining intramuscular injections of antagomir-22-3p (Greene et al., 2023).

## 4 Discussion

Nutrient restriction (60% of NRC) during late gestation altered ewe body weight gain and reduced body condition scores in this study. Nutrient-restricted ewes did not gain body weight during the last trimester and lost 0.8 units of body condition. Over 75% of fetal growth occurs during the last trimester of gestation in the sheep (Rattray et al., 1974), and maternal nutrient restriction during this time can influence fetal growth and muscle development (Reed et al., 2014; Hoffman et al., 2016). However, lamb birth weight was not affected in this study, possibly due to the use of mature, multiparous ewes. Changes in nutrient supply to gestating ewes alter muscle development and growth depending on when the restriction is applied in relation to muscle fiber development, the extent of nutrient restriction imposed, and the status of the ewe. However, even when birth weights are not reduced, alterations in muscle development are observed that impact postnatal muscle mass and carcass quality (Hoffman et al., 2017).

The use of antagomir technologies to reduce miR-22-3p expression in skeletal muscle was monitored by measuring circulating cell-free miR-22-3p levels to examine detectable changes in the bloodstream. On d 14 post-injection, miR-22-3p expression was downregulated in NR lambs injected with ANT22. On d 21 post-injection, miR-22-3p expression tended to be downregulated in NR lambs treated with ANT22. We followed the strategy described by Krützfeldt et al. (2005), administering three consecutive injections of antagomir, with an estimated inhibition period of 23 days post-injection, as demonstrated in mice. Our results indicate that relative expression levels of circulating miRNA can be monitored to assess the efficacy of treatment. The greater reduction in expression on d 14 may indicate that this was the time when the greatest difference occurred and that it may influence the need to re-inject at 14-day intervals instead of 24 days, as presented by Krützfeldt et al. (2005). Others have used antagomir-22-3p (15 mg/kg BW) administered via subcutaneous injections on days 0, 2, and 4 during the first week, followed by weekly injections for a total of 12 weeks in C57BL/6 male mice fed a high-fat (60% fat) diet (Thibonnier et al., 2020). They found that the antagomir-22-3p treatment reduced body weight gain and inguinal fat pad mass starting at 11 days, which persisted through 85 days on treatment. In addition, they reported a significant reduction in liver steatosis at 3 months after active treatment, indicating long-term effects that persist after weekly injections for 12 weeks. Cerro-Herreros et al. (2020) used antagomir-23b (12.5 mg/kg BW) administered subcutaneously or intravenously (tail vein) in mice to treat myotonic dystrophy. They found that intravenous injections upregulate Mbnl1, a target of miR-23b, to a greater extent than subcutaneous injection. In the second experiment, they examined the dose level (3, 12.5, or 40 mg/kg) via subcutaneous injection and reported that miR-23b was downregulated at 12.5 and 40 mg/kg levels in gastrocnemius and quadriceps muscle, respectively. In the third study, they evaluated the effects of a single dose (12.5 mg/kg BW) and found the greatest decrease in myotonia at 4 days post-injection, with differences still observed at 45 days post-injection. In mice, antagomir treatments are given at a higher dose level (12.5–15 mg/kg BW) on a body-weight basis, which is related to the differences in body size between the lamb (9 kg) and the mouse (38 g; Thibonnier et al., 2020; Cerro-Herreros et al., 2020). In our study, the dose level of antagomir-22-3p was 440 µg/lamb or 48.9 µg/kg BW to account for differences in animal size, and the treatment was administered intravenously into the lateral saphenous vein to target muscles of the leg.

For the ST muscle, samples were removed from the right (treated) and left (non-treated) ST of the NR-ANT22 lambs and the right ST of the CON-SHAM lambs to examine differences in the expression of miR-22-3p related to antagomir treatments versus the non-treated side in the same lamb and the control lambs. In the ST muscle, miR-22-3p expression was more downregulated (P < 0.05) in NR-ANT right ST (treated) compared to that in NR-ANT22_L ST (non-treated) and CON-SHAM_R ST. miR-22-3p expression did not differ between CON-SHAM_R and NR-ANT22_L ST (non-treated), indicating that antagomir-22-3p treatment downregulated miR-22-3p regardless of ewe treatment (NR or SHAM). Several other leg muscles were dissected from the right (treated) legs of NR-ANT22 and CON-SHAM lambs. miR-22-3p expression was examined in those muscles closest to the injection site (lateral saphenous vein) and the heart to evaluate whether systemic effects were elicited. miR-22-3p expression was downregulated in the semimembranosus and heart muscle at 24 days post-injection for NR-ANT22. miR-22-3p expression was not altered in the gastrocnemius muscles, which would be below the injection site in the lamb. The use of the blood flow resistance band on the right leg was designed to help with greater uptake of the antagomir into the plantar muscle group (Hagstrom et al., 2004; Teleni and Annison, 1986). The semimembranosus muscle is the largest in the hind leg of sheep and showed a significant downregulation of miR-22-3p expression. The heart muscle also showed a significant downregulation of miR-22-3p, indicating that the antagomir-22-3p injection influenced leg muscles closest to the injection site (SM and ST) but also traveled back to the heart. Hagstrom et al. (2004) also found that muscles closest to the injection site had the greatest positive staining cells for transgene expression tested in their study. Our results suggest that a more restrictive tourniquet on the leg, as used by Hagstrom et al. (2004), may be more effective than the blood flow resistance band used in this study for enhancing uptake into the leg muscles and reducing the likelihood of systemic return to the heart muscle. Individual muscle mass did not differ between the treatments, indicating that this short-term study did not enlarge the muscle fiber area or mass even though miR-22-3p was downregulated in specific muscles 24 days after injection.

MiR-22-3p is predicted to target HDAC4 and SIRT1 using multiple prediction software programs (Greene et al., 2023). These software prediction systems are only available for humans, rodents, and cattle, which makes it challenging to determine miRNA targets in the ovine. For this reason, we chose to examine several members of the HDAC family to evaluate changes in the gene expression of this important family involved in regulating muscle metabolism. Class I HDAC (HDAC1, 2, and 3) members were all downregulated with NR-ANT22 treatment. Class I HDAC members are reported to regulate metabolism in skeletal muscle (Molinari et al., 2023). Class II HDAC (HDAC4, 5, 6, 7, and 9) members were all downregulated in NR-ANT22 treatment, except for HDAC5, which was upregulated. Class II HDAC members are involved in the activation of the oxidative fiber phenotype (Potthoff et al., 2007; Molinari et al., 2023), and degradation of HDAC4 activates MEF2 to convert myofibers to a more oxidative state (Potthoff et al., 2007; Cohen et al., 2015). Class III HDAC members or the sirtuins (SIRT1, 3, and 6) were downregulated, except for SIRT3, which remained unchanged. SIRT1 and SIRT6 are both involved in regulating oxidative capacity in skeletal muscle (Potthoff et al., 2007; Philp et al., 2011; Molinari et al., 2023). Class IV HDAC (HDAC11) was also downregulated with NR-ANT22 treatment. HDAC11 deletion shifts skeletal muscle fibers to a more oxidative state (Hurtado et al., 2021). Overall, antagomir-22-3p treatment, whether administered directly into the muscle (Greene et al., 2023) or intravenously as in this study, produces a shift in myofibers to a more oxidative state, which is regulated by HDAC and SIRT expressions.

The NR-ANT22 treatment increased the number of muscle fibers in types I, IIa, and IIax fiber types, indicating more oxidative metabolism. Greene et al. (2023) also observed that the direct injection of NR-ANT22 in lamb longissimus muscle increased the transition of muscle fibers to a more oxidative state. Similarly, Wen et al. (2021) noted that miR-22-3p inhibition in C2C12 muscle cells promoted the conversion of fiber types toward oxidation via the AMPK/SIRT1/PGC1a pathway. These authors also reported that resveratrol inhibits miR-22-3p expression in C2C12 muscle cells and promotes the shift of muscle fibers toward oxidative metabolism (Huang et al., 2020; Wen et al., 2020; Wen et al., 2021). Ying et al. (2016) found that the overexpression of PGC1a in pigs increased oxidative muscle fibers and differences in gene expressions of myostatin (MSTN), myogenin (MyoG), and forkhead transcription factor 1 (FOXO1). Greater oxidative metabolism with miR-22-3p inhibition appears to be associated with the demands for protein synthesis and degradation capacity in the muscle fiber (van Wessel et al., 2010). Others reported a muscle paradox, where muscle fiber size and oxidative capacity are negatively related (Lynch and Koopman, 2019; van Wessel et al., 2010). However, Scheffler et al. (2014) reported that oxidative capacity and muscle hypertrophy can both increase in pig muscles with RyR1 and AMPKγ3 mutations. The increase in oxidative metabolism can help reduce energetic defects and maintain muscle health to support hypertrophy in long-term finishing studies (Scheffler et al., 2014). These results suggest that antagomir treatment alters muscle fiber metabolism, shifting it toward a more oxidative status, potentially through changes in HDAC family mRNA expression that regulate muscle fiber oxidative capacity. This study was a short-term study to examine changes in miRNA expression, and longer-term studies are needed to assess whether the changes in oxidative capacity and greater protein synthesis can lead to increased muscle hypertrophy and mass with antagomir treatments. Additional research is needed to further refine miRNA treatment injections to examine dose levels, multiple doses, and intravenous delivery methods (tourniquet) over a longer study period to examine changes in molecular and cellular effects of miR-22-3p inhibition and muscle fiber hypertrophy.

## Data Availability

The raw data supporting the conclusions of this article will be made available by the authors, without undue reservation.
